# Development of a multicomponent intervention to improve medication error reporting among healthcare professionals: a theory-informed approach using the behaviour change wheel and the theoretical domains framework in China

**DOI:** 10.3389/fphar.2026.1783200

**Published:** 2026-05-13

**Authors:** Hui Guo, Qian Guo, Ze Wang, Haixiong Wang

**Affiliations:** 1 Department of Pharmacy, Shanxi Cardiovascular Hospital, Taiyuan, Shanxi, China; 2 Department of Pharmacy, Cardiovascular Hospital Affiliated to Shanxi Medical University, Taiyuan, Shanxi, China; 3 Department of Pharmacy, Shanxi Key Laboratory of Heart Failure Precision Medicine, Taiyuan, Shanxi, China; 4 Department of Pharmacy, Second Hospital of Shanxi Medical University, Taiyuan, Shanxi, China

**Keywords:** behavior change wheel, behaviour change techniques, COM-B, incident reporting, medication error, patient safety, theoretical domains framework

## Abstract

**Introduction:**

Under-reporting of medication errors weakens reporting-and-learning systems and delays prevention efforts. Although medication error reporting is recognised as a core component of patient safety and organisational learning, reporting remains suboptimal in hospital practice. Healthcare professionals are central to the identification and reporting of medication errors and near misses; improving reporting therefore requires a change in the behaviour of clinical teams. In this paper, we describe the development of a theory- and evidence-informed multicomponent intervention, the Medication Error Reporting Enhancement Program (MERP), to promote medication error reporting in Chinese hospitals.

**Methods:**

A structured intervention development process guided by the Behaviour Change Wheel (BCW) was undertaken. It comprised three stages: (i) understanding the target behaviour by mapping determinants of medication error reporting identified in a Theoretical Domains Framework (TDF)-guided qualitative interview study to Capability-Opportunity-Motivation Behavior Model (COM-B) components; (ii) identifying intervention options by selecting BCW intervention functions and supporting policy categories using APEASE (Acceptability, Practicability, Effectiveness, Affordability, Side-effects and Equity); and (iii) specifying intervention content and delivery, including selecting behaviour change techniques (BCTs) using BCT Taxonomy v1 (BCTTv1), refining components through a stakeholder co-design workshop, and documenting the final programme using the Template for Intervention Description and Replication (TIDieR) checklist.

**Results:**

The behavioral diagnosis identified determinants of medication error reporting spanning all 14 TDF domains, with eight domains emerging as priority targets for change. Mapping these determinants to the BCW and applying the Affordability, Practicality, Effectiveness and Cost-Effectiveness, Acceptability, Side Effects/Safety, and Equality (APEASE) criteria resulted in an intervention architecture comprising seven intervention functions (education, training, persuasion, incentivisation, environmental restructuring, modelling and enablement) and four policy categories (communication/marketing, regulation, service provision and fiscal measures—conditional on resources). Intervention content was then specified using 14 BCTs, which were operationalised into the MERP and documented using the TIDieR checklist to enhance transparency and replicability.

**Conclusion:**

A TDF-to-BCW pipeline produced a theoretically grounded, system-aligned intervention blueprint that targets multi-level determinants of reporting behaviour. The resulting MERP offers a transparent basis for pilot testing and effectiveness-implementation evaluation. Its potential transferability to other safety-reporting behaviors is theoretical and mechanism-based, and should be empirically tested in other contexts.

## Introduction

Medication errors, defined by the US National Coordinating Council for Medication Error Reporting and Prevention (NCCMERP) as “any preventable event that may cause or lead to inappropriate medication use or patient harm”, remain a major and largely avoidable threat to patient safety (Merp, 2015). Globally, around 1 in 10 patients experience harm during healthcare, and more than half of patient harm is considered preventable; importantly, a substantial proportion of preventable harm is medication-related (Slawomirski, 2020; Panagioti et al., 2019). Medication errors also impose a considerable economic burden on health systems, with global costs estimated at US$42 billion annually (Tariq et al., 2025).

Medication error reporting and learning systems are essential for detecting risks, understanding contributory factors, and supporting organisational learning and prevention (Network, 2023; Elden and Ismail, 2016). In hospital and community settings, voluntary reporting can help identify system weaknesses and inform corrective actions to reduce future harm (Adie et al., 2021). However, under-reporting remains common. In China, although medication safety reporting has been incorporated into policy and hospital quality management requirements, national data still suggest substantial implementation gaps (Zhang et al., 2024; Bai et al., 2025).

Previous studies suggest that medication error reporting can be improved through changes in reporting systems, workflow, and safety culture. Interventions such as electronic reporting tools, standardised reporting processes (Stump, 2000; Savage et al., 2005), staff education, feedback, and non-punitive safety initiatives have been associated with increased reporting in some settings (Yousef et al., 2021; Wat et al., 2025). Effects appear greater when interventions are multicomponent and address both individual and organisational determinants (Force et al., 2006; Lehmann et al., 2007; Yousef et al., 2021).

Pharmacists have played important roles in many of these multicomponent interventions through education, reporting support, medication review, and workflow optimisation. Evidence suggests that pharmacist involvement can substantially strengthen reporting performance and medication safety. For example,, clinical pharmacist-led intervention and education increased medication error reporting approximately six-fold in a paediatric intensive care unit and reduced serious medication errors (Costello et al., 2007). In a hospital emergency department, the number of reported medication errors increased 14.8-fold after the addition of two clinical pharmacists (Weant et al., 2010). In a retrospective review of the Malaysian national medication error reporting system, pharmacists were responsible for identifying and reporting 92.1% of medication errors (Samsiah et al., 2020). Pharmacist-led medication safety activities, including medication reconciliation, prescription review, reporting, and education, have also been associated with reductions in medication errors (Gillani et al., 2021).

However, important limitations remain in the existing intervention literature. Much of the existing literature consists of single-site before-after studies with heterogeneous outcomes, limiting comparability and making it difficult to identify which components drive change. In addition, although pharmacists are often involved, many interventions are not explicitly theory-informed and their active ingredients are poorly specified. This limits reproducibility, transferability, and implementation.

A more systematic and theory-driven approach to intervention development is therefore needed. Behavioral studies on medication error reporting have used frameworks such as the Theory of Planned Behavior (Natan et al., 2017), Knowledge-Attitudes-Practice (Yousef et al., 2021), and the Theoretical Domains Framework (TDF) to identify barriers and facilitators. The TDF provides a comprehensive approach to behavioral diagnosis, while the BCW offers a structured method for selecting intervention functions and policy categories (Michie et al., 2011; Michie et al., 2014). Behaviour Change Technique Taxonomy v1 (BCTTv1) can then be used to specify active ingredients, and the Template for Intervention Description and Replication (TIDieR) can improve reporting transparency and replicability.

For example, Stewart et al. identified fear of repercussions, concerns about confidentiality, and perceived impacts on career progression as key barriers to medication error reporting in Qatar (Stewart et al., 2018), while Alqubaisi et al. highlighted lack of feedback and time constraints in the United Arab Emirates (Alqubaisi et al., 2016). Building on such a diagnosis, the BCW offers a transparent pathway to select appropriate intervention functions and translate them into clearly specified Behaviour Change Techniques (BCTs), thereby improving the precision and replicability of intervention design (Michie et al., 2013; Hoffmann et al., 2014). Used sequentially, these frameworks provide a coherent pathway from behavioral diagnosis to intervention specification.

Such end-to-end, theory-informed intervention development remains uncommon in the medication error reporting field, particularly in Chinese hospitals. This is important because reporting behaviour is shaped not only by knowledge and skills (Stewart et al., 2018), but also by workload pressure, organisational hierarchy (Hu et al., 2024), social influences, and concerns about blame and reputational consequences (Hu et al., 2024). Interventions imported directly from other settings may therefore have limited acceptability or feasibility. A locally adapted, theory-informed, and clearly specified intervention is needed.

Accordingly, this study aimed to develop a feasible and replicable pharmacist-led multicomponent intervention, the Medication Error Reporting Enhancement Program (MERP), to improve medication error reporting among Chinese healthcare professionals. The objectives were to: (i) identify key modifiable determinants of medication error reporting using a COM-B/TDF-informed behavioral diagnosis; (ii) map these determinants to BCW intervention functions and policy categories, and specify active ingredients using BCTTv1 terminology; and (iii) describe MERP in sufficient detail for replication and future evaluation using the TIDieR framework.

## Methods

### Study design

**FIGURE 1:**
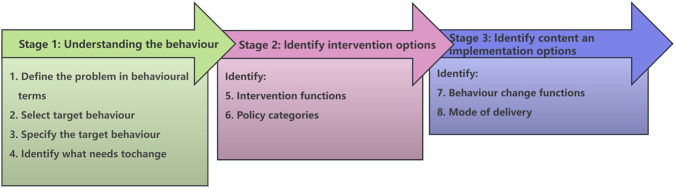
Stages involved in the development of an intervention using the BCW Adapted with permission from Michie et al. (2014), Copyright © Susan Michie, Lou Atkins and Robert West 2014.

This was a theory-informed intervention development study aimed at designing and specifying a multicomponent programme to improve medication error reporting among healthcare professionals in Chinese hospitals. Guided by the BCW, the study followed three stages (Figure 1): understanding the behaviour, identifying intervention options, and specifying intervention content and implementation procedures (Michie et al., 2014). The purpose was to develop a transparent, context-appropriate, and reproducibly specified intervention blueprint, rather than to evaluate intervention effectiveness.

Intervention development drew on three sources: our previously published TDF-guided qualitative interview study on determinants of medication error reporting in China (Guo et al., 2025), relevant literature, and stakeholder input from a co-design workshop. The TDF and COM-B model were used for behavioral diagnosis, the BCW for intervention-function and policy-category selection, BCTTv1 for specifying active ingredients, and TIDieR for describing delivery and implementation parameters. Two authors (HG and QG) independently undertook the main mapping and appraisal steps; discrepancies were resolved by discussion and, where needed, by adjudication from the senior author (ZW). Components were retained based on theoretical fit, contextual feasibility, acceptability, and non-redundancy. The final output was the Medication Error Reporting Enhancement Program (MERP), a pharmacist-led multicomponent intervention.

#### Stage 1: understanding the behaviour (behavioral diagnosis)

Stage 1 comprised a four-step behavioral diagnosis to define the reporting problem in Chinese hospitals and identify modifiable targets for change. First, we synthesised available evidence to characterise the behavioral problem as persistent under-reporting of medication errors (Step 1). We then specified and decomposed medication error reporting into observable behavioral components, including recognising reportable events, deciding to report, and completing and submitting reports within routine workflow, and selected the priority target behaviour for intervention development as timely reporting of medication errors and near misses by healthcare professionals in Chinese hospitals (Steps 2–3). Determinants influencing this behaviour were examined using our TDF-guided qualitative interview study of Chinese healthcare professionals (Step 4) (Guo et al., 2025).

Two authors reviewed the identified determinants and mapped them to TDF domains and COM-B components using published guidance. Where determinants could plausibly fit more than one COM-B category, classification was based on their primary behavioral role in the reporting context. Priority domains for intervention development were selected based on salience in the qualitative data, plausibility of modification, and relevance to the target behaviour. This process clarified what needed to change to improve medication error reporting.

#### Stage 2: identifying intervention options

Stage 2 focused on identifying intervention options using the BCW approach and included two linked steps: selecting candidate intervention functions and then specifying enabling policy categories. First, candidate intervention functions were identified by linking priority behavioral determinants to BCW guidance and published TDF–BCW mapping work (Step 5) (Michie et al., 2011; Michie et al., 2014). These candidate functions were appraised in relation to routine clinical workflows, existing medication safety structures, and the wider organisational and medico-legal context in Chinese hospitals. Options were screened using the APEASE criteria (Acceptability, Practicability, Effectiveness, Affordability, Side-effects, and Equity). Second, we identified policy categories that could support implementation of the selected intervention functions in Chinese hospitals (Step 6). Policy categories were assessed using the same general principles, including theoretical relevance, feasibility, acceptability, and likely implementation value, alongside APEASE considerations. Selection did not rely on APEASE alone; candidate intervention functions and policy categories were also considered in terms of their conceptual linkage to priority determinants, likely acceptability in a sensitive reporting context, feasibility in routine practice, and whether they added distinct value to the intervention package.

Two authors (HG and QG) independently appraised candidate intervention functions and policy categories using a structured checklist. Brief justifications for inclusion, exclusion, or conditional retention were recorded, and discrepancies were resolved through discussion and consensus with the senior author (ZW). The retained intervention functions and policy categories (Tables 2–4) were then taken forward for intervention specification.

#### Stage 3: identifying content and implementation options

Stage 3 focused on specifying MERP content and delivery and comprised two core steps: selecting BCTs and defining delivery modes and key implementation parameters.

First, First, a candidate list of BCTs was generated based on the selected intervention functions, priority behavioral determinants, theory-to-technique mapping, and relevant literature on medication error reporting, medication safety, and related safety-culture interventions (Step 7) (Michie et al., 2013). Candidate BCTs were then appraised using APEASE, with particular attention to practicality, acceptability, and feasibility in busy hospital settings. BCT selection was further guided by whether each technique addressed a clear behavioral mechanism, could be delivered within a pharmacist-led model, and added value beyond other retained techniques. BCTs were excluded when they were overly burdensome, difficult to sustain in routine practice, conceptually duplicative, or likely to create undesirable consequences such as excessive reporting pressure or target-driven behaviour.

Second, intervention delivery and key implementation parameters were specified using TIDieR (Step 8) (Michie et al., 2014), including what would be delivered, how and by whom it would be delivered, and when and how often it would occur. The retained BCTs were operationalised into intervention components suitable for Chinese hospitals. Two authors (HG and QG) independently reviewed and shortlisted candidate BCTs, and final decisions were reached through discussion and, where needed, adjudication by the senior author (ZW).

### Stakeholder co-design workshop

To improve contextual fit and implementation readiness, we conducted a stakeholder co-design workshop during Stage 3. Stakeholders were purposively sampled to represent pharmacy, medicine, nursing, quality/safety management, and information technology. Participants reviewed the draft COM-B/TDF mapping, APEASE shortlists, and candidate BCT list in advance. The workshop was used to review proposed intervention functions, policy supports, and BCTs; assess feasibility and acceptability within local workflow constraints; and refine delivery details and implementation supports in line with TIDieR. Key refinements included clarifying pharmacist-led coordination roles, simplifying reporting guidance and materials, and optimising prompts/cues and workflow integration. A total of 23 stakeholders participated, including pharmacists (n = 5), physicians (n = 6), nurses (n = 5), quality/safety personnel (n = 5), and IT staff (n = 2). Outputs from the workshop were used to finalise MERP content and delivery specifications.

### Ethics considerations

This manuscript reports an intervention-development blueprint. No new patient-level clinical data were collected for this paper. The behavioral diagnosis was informed by our previously published TDF-guided qualitative interview study (Guo et al., 2025), which received ethical approval from the Ethics Committee of the Second Hospital of Shanxi Medical University (Ref no: 2023-YX-202) and obtained informed consent from participants. The stakeholder co-design workshop involved healthcare staff to refine feasibility and delivery details; no identifiable personal data are reported in this manuscript.

## Results

Following behavioral diagnosis, theory-informed mapping, structured appraisal, and stakeholder refinement, candidate intervention functions, policy categories, and BCTs were progressively shortlisted and integrated into the MERP. The overall process is summarised in Figure 1. In brief, the TDF/COM-B analysis was used to identify what needed to change, the BCW was used to determine how change could be supported at the level of intervention functions and policy categories, BCTTv1 was then used to specify the active ingredients of the intervention, and TIDieR was used to organise these elements into a deliverable intervention protocol. This sequential process resulted in a pharmacist-led, multicomponent intervention that linked behavioral determinants to clearly specified content and delivery procedures.

### Stage 1: understanding the behaviour

#### Step 1: define the problem in behavioral terms

The behavioral problem was defined as persistent under-reporting of medication errors and near misses by healthcare professionals in Chinese hospitals. Drawing on the literature and our previous TDF-guided qualitative study, under-reporting was characterised not simply as a lack of reporting opportunity, but as a behaviour influenced by multiple interacting determinants, including limited reporting knowledge, uncertainty about reportable events, time pressure, workflow burden, social and hierarchical constraints, and concerns about blame, disputes, and reputational consequences. This framing established medication error reporting as a behavioral target requiring a multilevel intervention rather than a single educational or procedural response.

#### Step 2: select the target behaviour

Among the interrelated behaviors involved in medication error reporting, the priority target behaviour was selected as timely reporting of medication errors and near misses by physicians, nurses, and pharmacists in Chinese hospitals. This behaviour was prioritised because it was central to strengthening reporting-and-learning systems, directly relevant to medication safety improvement, and amenable to change through intervention.

#### Step 3: specify the behaviour

To increase precision, the target behaviour was specified in actionable terms in line with BCW guidance (Michie et al., 2014). Healthcare professionals were expected to: report a medication error or near-miss whenever identified during routine work (when), across inpatient, outpatient, and pharmacy settings along the medication-use process (where), as part of their everyday clinical workflow and in coordination with relevant colleagues as required (with whom), and consistently for each eligible event (how often). In addition, behavioral specification explicitly considered context-relevant influences, including workload constraints, organisational hierarchies, and concerns regarding blame, disputes, and reputational risk.

#### Step 4: identify what needs to change

Behavioral determinants identified in our prior qualitative study (Guo et al., 2025) were reviewed, grouped into TDF domains, and mapped to COM-B components. Influences spanning all 14 TDF domains were identified, confirming that medication error reporting is a multidimensional behaviour shaped by capability, opportunity, and motivation. However, eight domains emerged as the most salient and modifiable barriers: Knowledge, Skills, Beliefs about capabilities, Beliefs about consequences, Memory, attention and decision processes, Environmental context and resources, Social influences, and Emotion. These domains were prioritised because they were prominent in the qualitative data, directly relevant to the target behaviour, and judged to be amenable to intervention.

Taken together, these findings clarified what needed to change. In terms of capability, healthcare professionals required greater understanding of reportable events and more confidence and skill in completing reports. In terms of opportunity, reporting needed to be easier to access, less burdensome, and better supported by workflow and colleagues. In terms of motivation, staff needed to view reporting as worthwhile, feasible, professionally legitimate, and psychologically safe. The mapping of COM-B/TDF domains to “what needs to change” is summarised in Table 1.

**TABLE 1:** Intervention functions for improving healthcare professionals’ medication error reporting, identified using COM-B and TDF.

COM-B component	TDF domain	What needs to change	Intervention function(s)	Illustrations
Capability	Knowledge	Ensure HCPs understand what constitutes a medication error (ME) and are familiar with reporting rules and procedures	Education	ME definitions/classifications; reporting guidance via training sessions, manuals, quick-reference cards, and online modules
Capability	Skills	Strengthen practical skills to identify, document, and submit ME reports accurately and efficiently	Training	Scenario-based practice, case discussions, simulation drills, hands-on training on the reporting platform
Opportunity	Environmental context and resources	Reduce time/effort barriers by improving access, usability, and workflow integration; ensure resources are available	Environmental restructuring	Optimise the electronic reporting system (simplified forms, templates, barcode/EMR linkage), mobile access, streamline steps
Opportunity	Social influences	Create a supportive social environment where leaders and peers encourage reporting and model speaking up	Enablement; modelling; training	Champions/role models; team debriefs; peer support; leadership endorsement; interdisciplinary safety huddles
Motivation	Social/professional role and identity	Clarify that reporting is a professional responsibility aligned with patient safety roles	Education; training	Embed expectations in job descriptions and department policies; clarify responsibilities and accountability
Motivation	Beliefs about capabilities	Increase confidence in identifying/reporting correctly and that the process is manageable	Training; enablement	Step-by-step guidance, coaching, walk-through support; helpdesk/medication safety officer support
Motivation	Beliefs about consequences	Increase perceived benefits and reduce fear of negative consequences	Persuasion	Communicate learning and improvements from reporting; reinforce non-punitive principles and confidentiality
Motivation	Reinforcement	Provide positive reinforcement to sustain reporting behaviors	Incentivisation	Recognition programmes, feedback dashboards, certificates, department-level awards for safety contributions
Motivation	Emotion	Reduce anxiety/fear (blame, litigation, reputation risk) associated with reporting	Enablement; persuasion	Promote psychological safety; anonymous/just-culture options; explicit protection statements; supportive messaging
Motivation	Goals	Encourage departments/individuals to set safety/reporting goals and track progress	Enablement; incentivisation	Set reporting improvement goals; include indicators in quality targets; provide progress feedback
Motivation	Intentions	Translate intention into action by lowering friction and strengthening commitment	Enablement; environmental restructuring	One-click reporting entry points; workflow-integrated prompts; team commitment statements
Motivation	Memory, attention and decision processes	Increase salience and cueing so HCPs remember to report when events occur	Environmental restructuring; enablement	Prompts/cues at points of care; pop-up reminders; checklists and quick links
Motivation	Behavioral regulation	Support routine reporting habits and self-regulation	Enablement; training	Ongoing reinforcement, checklists, periodic refreshers; audit and feedback on reporting quality
Motivation	Optimism	Increase optimism that reporting leads to improvement and is worthwhile	Persuasion	Share visible improvements and ‘you reported—here’s what changed’ messages

COM-B, capability, Opportunity, Motivation; TDF, theoretical domains framework; HCPs, healthcare professionals. Intervention function definitions follow the Behaviour Change Wheel guide. APEASE, acceptability, Practicability, Effectiveness, Affordability, Side-effects, Equity.

### Stage 2: identifying intervention options

#### Step 5: identify intervention functions

Based on the COM-B/TDF behavioral diagnosis, candidate BCW intervention functions were identified and appraised for theoretical fit, feasibility, acceptability, and risk of unintended consequences, followed by APEASE screening. Seven of the nine BCW intervention functions were retained: Education, Training, Persuasion, Education and Training were retained because they directly addressed deficits in knowledge and skills related to identifying and reporting medication errors. Persuasion was retained because beliefs about consequences and emotional barriers indicated a need to reframe reporting as a positive, learning-oriented activity. Environmental restructuring and Enablement were retained because workflow barriers, limited time, and procedural complexity were important contextual obstacles. Modelling was retained because social influences and hierarchical norms suggested value in visible role models and local champions. Incentivisation was retained cautiously because reinforcement could help sustain reporting, but only if used in a way that supported learning rather than simple report-counting.

By contrast, Coercion and Restriction were excluded. These options were considered poorly suited to a sensitive reporting environment and were judged likely to increase fear, blame, or resistance rather than promote reporting. The APEASE appraisal and final selection of intervention functions are shown in Table 2.

**TABLE 2:** Selection of intervention functions using APEASE.

Intervention function	APEASE consideration	Rationale (summary)	Selected
Education	Affordable; practical; acceptable; low risk; equitable	Improves knowledge and correct understanding of medication error reporting	Yes
Training	Practical; acceptable; manageable cost	Builds reporting-related skills and confidence	Yes
Persuasion	Low resource; acceptable if framed as learning	Shifts attitudes and perceived consequences; supports motivation	Yes
Incentivisation	Feasible if low-cost recognition	Reinforces reporting and learning; safeguards needed to avoid quantity over quality	Yes (with caution)
Environmental restructuring	Feasible with workflow/platform changes	Reduces time/effort barriers and integrates reporting into work processes	Yes
Modelling	Feasible via champions/role models	Normalises reporting and supports speak-up culture	Yes
Enablement	Feasible through barrier reduction and supports	Provides practical help (support roles, feedback, facilitation)	Yes
Coercion	Low acceptability; potential adverse effects	May increase fear/blame and discourage reporting	No
Restriction	Poor fit and feasibility	Not appropriate for reporting behaviour; may undermine safety culture	No

APEASE, acceptability, Practicability, Effectiveness, Affordability, Side-effects, Equity.

#### Step 6: identify policy categories

BCW policy categories were then reviewed as higher-level supports for implementation of the selected intervention functions. Four categories were retained as feasible and context-appropriate: Communication/marketing, Regulation, Service provision, and Fiscal measures (conditional on funding). Communication/marketing was retained to support awareness-building and non-punitive messaging; Regulation was retained to clarify responsibilities, procedures, and protections; Service provision was retained because practical support structures were needed to embed reporting support into routine work; and Fiscal measures were retained conditionally as a possible means of supporting recognition or modest implementation resources.

Other categories were not prioritised. Guidelines were not retained as a stand-alone category because existing rules alone were unlikely to change behaviour. Legislation was beyond hospital-level control, and Environmental/social planning was considered less directly actionable in the current setting. The APEASE appraisal and final policy-category selection are presented in Table 3.

**TABLE 3:** Selection of policy categories using APEASE.

Policy category	APEASE consideration	Rationale (summary)	Selected
Communication/marketing	Affordable; practical; acceptable	Supports awareness, norms, and non-punitive messaging via internal communication channels	Yes
Regulation	Practical within hospital governance	Supports protection of reporters, accountability, and standardised processes	Yes
Fiscal measures	Budget dependent	Potentially useful for recognition/resources; feasibility depends on funding	Yes (conditional)
Service provision	Practical; acceptable	Provides reporting support services and resources; embeds reporting into routine services	Yes
Guidelines	Limited added value alone	May overlap with existing rules; not prioritised as a standalone category	No
Legislation	Beyond hospital-level control	Not directly actionable at the study setting level	No
Environmental/social planning	Less directly actionable	Not prioritised within current implementation constraints	No

APEASE, acceptability, Practicability, Effectiveness, Affordability, Side-effects, Equity.

### Stage 3: identifying content and implementation options

#### Step 7: identify behaviour change techniques

To translate the selected intervention functions and policy supports into specific intervention content, candidate BCTs were identified using BCTTv1 (Michie et al., 2013). Guided by the COM-B/TDF diagnosis (Step 4) and the BCW selections (Steps 5–6), we compiled a long-list of potentially relevant BCTs and screened them against APEASE to ensure feasibility and acceptability in busy Chinese hospital settings. Following this appraisal, 14 BCTs were retained for inclusion in the medication error reporting programme (MERP) (Table 4). These comprised: Problem solving (BCTTv1 1.2), Action planning (BCTTv1 1.4), Feedback on outcome(s) of behaviour (BCTTv1 2.7), Social support (unspecified) (BCTTv1 3.1), Social support (practical) (BCTTv1 3.2), Instruction on how to perform the behaviour (BCTTv1 4.1), Information about health consequences (BCTTv1 5.1), Information about social and environmental consequences (BCTTv1 5.3), Demonstration of the behaviour (BCTTv1 6.1), Prompts/cues (BCTTv1 7.1), behavioral practice/rehearsal (BCTTv1 8.1), Credible source (BCTTv1 9.1), Restructuring the physical environment (BCTTv1 12.1), and Adding objects to the environment (BCTTv1 12.5) (Table 4).

**TABLE 4:** Selection of Behaviour Change Techniques (BCTs) using APEASE (BCTTv1 terminology).

BCT (BCTTv1)	Example application for MERP	Related function(s)	APEASE result	Selected
1.1 goal setting (behaviour)	Require each healthcare professional to report at least 10 medication errors per month to establish a behavioral target and gradually increase reporting volume	Enablement	Did not meet practicability	No
1.2 problem solving	Team discussions to identify and resolve practical difficulties in reporting (e.g., unclear procedures, time pressure). Delivered/led by clinical pharmacists as local medication safety champions	Enablement	Met all APEASE criteria	Yes
1.3 goal setting (outcome)	Set a target of reducing medication error incidents by 10%, track progress via the reporting system, and reward departments meeting the target.	Enablement	Did not meet practicability	No
1.4 action planning	Provide a “medication error reporting guide” specifying step-by-step actions so staff can act quickly when an error is identified	Enablement	Met all APEASE criteria	Yes
1.5 review behaviour goal(s)	Monthly review of whether staff achieved predefined reporting targets and adjust targets if needed	Enablement	Did not meet practicability	No
1.7 review outcome goal(s)	Periodically review whether outcome targets related to reporting have been achieved	Enablement	Did not meet practicability	No
2.1 monitoring of behaviour by others without evidence of feedback	Cross-department peer observation to check whether others report medication errors in a timely manner	Incentivisation	Did not meet practicability and acceptability	No
2.2 feedback on behaviour	Use the electronic system to provide timely feedback on types of reported errors and handling outcomes, plus thank-you letters or commendation emails	Education; persuasion; incentivisation; training	Did not meet practicability	No
2.3 self-monitoring of behaviour	System automatically logs each report; individuals review and summarise their monthly reporting frequency	Training; enablement; education; incentivisation	Did not meet practicability	No
2.5 monitoring outcome of behaviour by others without evidence of feedback	Leadership regularly analyses departmental reporting outcomes and error rates and adjusts policy accordingly	Incentivisation	Did not meet practicability	No
2.7 feedback on outcome(s) of behaviour	Provide feedback explaining how a report contributed to patient safety improvements or prevented future errors	Persuasion; education; training; incentivisation	Met all APEASE criteria	Yes
3.1 social support (unspecified)	Leaders and colleagues actively encourage and support staff to report medication errors	Enablement	Met all APEASE criteria	Yes
3.2 social support (practical)	Establish a “medication error reporting support group” to help colleagues resolve questions about the reporting process	Enablement	Met all APEASE criteria	Yes
4.1 instruction on how to perform the behaviour	Develop an operational manual detailing each step of reporting, including completing and submitting the report	Training	Met all APEASE criteria	Yes
5.1 information about health consequences	Regular case-based communications showing how reporting reduces patient harm and health risks	Education; persuasion	Met all APEASE criteria	Yes
5.3 information about social and environmental consequences	Communicate that reporting reduces medication waste and improves patient safety, generating broader social/system benefits	Persuasion; education	Met all APEASE criteria	Yes
6.1 demonstration of the behaviour	Senior staff demonstrate the reporting process during training sessions to ensure correct understanding and performance	Modelling; training	Met all APEASE criteria	Yes
7.1 prompts/cues	Posters/signage in dispensing areas or wards stating “if a medication error is identified, please report immediately.”	Education; environmental restructuring	Met all APEASE criteria	Yes
8.1 behavioral practice/rehearsal	Quarterly simulation drills of reporting scenarios to practise rapid and accurate reporting	Training	Met all APEASE criteria	Yes
9.1 credible source	Invite pharmacy experts/clinical researchers to share best practices and institutional support for reporting in internal meetings	Persuasion	Met all APEASE criteria	Yes
12.1 restructuring the physical environment	Optimise workspace layout/interface access so the reporting system is easier to reach and use	Environmental restructuring; enablement	Met all APEASE criteria	Yes
12.5 adding objects to the environment	Provide reporting forms/manuals/quick guides at workstations; add QR codes/quick links for rapid access	Environmental restructuring; enablement	Met all APEASE criteria	Yes

BCT, labels follow the Behaviour Change Technique Taxonomy v1 (BCTTv1). APEASE, acceptability, Practicability, Effectiveness, Affordability, Side-effects, Equity; MERP, Medication Error Reporting Enhancement Program. Restriction was not included. In this study, BCTs, were selected to change how people think, feel, or respond and to reduce practical barriers, rather than to impose external restrictions; therefore, no BCTTv1 techniques were mapped to the Restriction intervention function (Michie et al., 2014).

Several candidate BCTs were not retained. In general, excluded BCTs were those judged to be insufficiently feasible in routine hospital practice, overly burdensome, conceptually duplicative, or likely to produce undesirable consequences such as target-driven reporting, surveillance concerns, or excessive reporting pressure. For example, techniques requiring predefined individual reporting targets or intensive monitoring were not retained because they were considered to have limited practicability and risked shifting attention from reporting quality and learning to reporting volume. Thus, BCTTv1 was used not as a separate parallel framework, but as a means of specifying the active ingredients of the intervention functions already selected through the BCW process.

#### Step 8: mode of delivery (intervention specification)

The retained BCTs were synthesised into a pharmacist-led multicomponent intervention, the Medication Error Reporting Enhancement Program (MERP). MERP was designed as a primarily face-to-face, group-based intervention supported by written, visual, and environmental materials. Core materials included an expert consensus document, a structured training handbook or leaflets, and workstation posters and cue materials to prompt reporting at the point of care.

Using TIDieR, the intervention was then specified in terms of its rationale, materials, procedures, providers, mode of delivery, setting, schedule, and planned fidelity considerations (Hoffmann et al., 2014). The intervention was intended for delivery across medication-use settings in hospitals, including wards, outpatient areas, pharmacies, and training spaces. Pharmacists were positioned as the main coordinators of delivery, supported by multidisciplinary clinical leads, managers, and IT staff where needed. Tailoring, modifications, and actual fidelity were not applicable at this stage because MERP remained at the intervention-development stage. The full TIDieR-based description is provided in Table 5.

**TABLE 5:** TIDieR checklist (Template for Intervention Description and Replication) for MERP.

TIDieR item	Description	MERP (example content)
1. Brief name	Provide the name or a phrase describing the intervention	Medication error reporting enhancement program (MERP)
2. Why	Describe the rationale, theory, and/or goal of the essential elements of the intervention	MERP was developed using the BCW framework. It combines multi-level components to strengthen healthcare professionals’ capability, opportunity and motivation for medication error reporting, with the goal of reducing under-reporting and improving medication safety
3. What (materials)	Describe any physical or informational materials used, including those provided to participants or used in delivery/training; indicate where materials can be accessed (e.g., online appendix/URL)	Materials include: (1) expert consensus on medication error management in China; (2) an expert-developed training handbook on medication error reporting; (3) simulation scenarios and related equipment; (4) prompts/signage and cue cards. (Access route to be provided in appendix/online repository when implemented.)
4. What (procedures)	Describe the procedures, activities and/or processes used in the intervention, including any enabling/support activities	Delivery of the 14 selected BCTs to healthcare professionals, supported by training, simulation, prompts/cues, and optimisation of reporting workflow/system as needed
5. Who provided	Describe the expertise, background, and any specific training of intervention providers	A pharmacist-led medication safety team coordinates MERP. Core providers include (1) senior clinical pharmacists/medication-safety officer (content leadership, coaching and feedback), (2) multidisciplinary clinical leads (case discussion and local workflow alignment), (3) hospital managers (governance and just-culture reinforcement), and (4) IT support staff (e-reporting platform optimisation)
6. How	Describe the mode(s) of delivery (e.g., face-to-face, online, telephone) and whether delivered individually or in groups	Primarily face-to-face group delivery. The delivery mode for specific BCTs may vary depending on the component (e.g., on-site demonstration, digital reminders). The programme is pharmacist-led, with pharmacists facilitating group training and simulation and supporting point-of-care reporting
7. Where	Describe the type(s) of location(s) where the intervention occurs, including any necessary infrastructure/features	Hospital settings along the medication-use process: Pharmacy department and clinical training rooms (theory sessions); wards/outpatient areas/pharmacy (hands-on practice and simulation)
8. When and how much	Describe the number of sessions, schedule, duration, intensity, or dose	Week 1: Intensive training (2 h/session × 4 sessions). Weeks 2–4: Weekly simulation practice (1 h/session × 1 per week). From week 5: Continuous environmental prompts, plus refresher training quarterly
9. Tailoring	If planned to be personalised, titrated, adapted, or otherwise tailored, describe what, why, when, and how	Not applicable at the development stage (intervention not yet implemented)
10. Modifications	If modifications occurred during the study, describe what was modified, why, when, and how	Not applicable at the development stage (intervention not yet implemented)
11. How well (planned)	Describe planned assessment of fidelity/adherence, by whom, and strategies to maintain/improve fidelity	Fidelity will be monitored across four dimensions: Content delivered, delivery frequency, duration, and coverage/reach, with periodic checks and feedback to providers to maintain fidelity
12. How well (actual)	Describe the extent to which the intervention was delivered as planned (if assessed)	Not applicable at the development stage (intervention not yet implemented)

TIDieR, template for intervention description and replication; BCW, behaviour change wheel.

## Summary of the final intervention and implementation pathway

Overall, the intervention-development process resulted in a pharmacist-led, theory-informed, and context-adapted intervention blueprint. MERP addresses capability through education, skills training, behavioral practice, and clear reporting instructions; opportunity through workflow support, environmental restructuring, prompts/cues, and practical assistance; and motivation through persuasive messaging, credible sources, social support, feedback on outcomes, and cautious reinforcement.

The implementation pathway therefore proceeds sequentially from behavioral diagnosis (TDF/COM-B), to selection of intervention functions and policy supports (BCW), to specification of active ingredients (BCTTv1), and finally to intervention description and delivery planning (TIDieR). In this way, the different frameworks were synthesised into a single intervention protocol rather than used as parallel or overlapping methods. This process produced a transparent and reproducible blueprint for future piloting and evaluation of medication error reporting improvement in Chinese hospitals.

## Discussion

Using a structured, theory-informed intervention development process, this study developed pharmacist-led multicomponent intervention blueprint (MERP) to improve medication error/near-miss reporting among healthcare professionals in Chinese hospitals. Guided by the BCW and grounded in our TDF-informed qualitative evidence, we first clarified “what needs to change” across COM-B, identifying eight predominant barrier domains (Guo et al., 2025). We then translated these determinants into a feasible intervention architecture using APEASE screening, retaining seven intervention functions and four supporting policy categories. Finally, we operationalised the intervention content into 14 BCTs using BCTTv1 and specified MERP delivery using TIDieR, enhancing transparency and replicability. Rather than assembling a pragmatic bundle without clear logic, this process linked identified behavioral determinants to explicit intervention content and implementation arrangements. In doing so, MERP addresses a common limitation in the medication error reporting literature, where interventions have often been multicomponent but insufficiently theory-informed or poorly specified.

Overall, MERP aligns with a consistent pattern in the medication error reporting literature: reporting is more likely to improve when interventions move beyond stand-alone training and address multiple interacting determinants—capability gaps, workflow friction, social/organisational norms, and reinforcement loops. In previous before–after evaluations, introducing new reporting systems and standardising processes was frequently associated with increased reporting, with larger gains when combined with multi-component strategies such as non-punitive culture-building, dedicated safety roles/teams, education/training, and reinforcement mechanisms (Force et al., 2006; Abstoss et al., 2011; Ramírez et al., 2018). Our study extends this literature by making the behavioral logic of such multicomponent approaches more explicit. In MERP, the different components were not selected as isolated activities, but as linked responses to capability, opportunity, and motivation barriers identified in the behavioral diagnosis.

Education and Training were retained because they directly address deficits in reporting knowledge and practical skills. In the present context, healthcare professionals may be uncertain about what constitutes a reportable medication error or near miss and may lack confidence in navigating reporting procedures. These functions were translated into specific techniques including Instruction on how to perform the behaviour (BCTTv1 4.1; Table 4), Demonstration of the behaviour (BCTTv1 6.1; Table 4), and behavioral practice/rehearsal (BCTTv1 8.1; Table 4). Together, these techniques are intended to strengthen procedural competence and reporting confidence. This is consistent with previous studies showing that practical guidance and repeated exposure to reporting procedures can improve engagement with reporting systems (Stump, 2000; Force et al., 2006).

Persuasion and Modelling were also important because reporting behaviour is shaped not only by procedural knowledge, but also by perceived consequences, professional norms, and emotional responses. In settings where fear of blame, hierarchical culture, and concern about professional reputation discourage speaking up, reporting may be viewed as risky rather than constructive (Stewart et al., 2018; Gao et al., 2024). MERP therefore incorporates Credible source (BCTTv1 9.1; Table 4), Information about health consequences (BCTTv1 5.1; Table 4), Information about social and environmental consequences (BCTTv1 5.3; Table 4), and Social support (unspecified) (BCTTv1 3.1; Table 4) to reframe reporting as a professional, learning-oriented, and safety-enhancing practice. Modelling is particularly relevant because visible support from respected colleagues or local champions may help normalise reporting and signal that it is acceptable and valued. This is in line with previous observations that leadership endorsement and role modelling can shape safety culture and encourage reporting (Guan et al., 2017).

Environmental restructuring and Enablement were retained because the behavioral diagnosis highlighted substantial “last-mile” barriers in routine practice, including time pressure, reporting inconvenience, competing demands on attention, and uncertainty about how to act when an event occurs. MERP addresses these issues through Prompts/cues (BCTTv1 7.1; Table 4), Restructuring the physical environment (BCTTv1 12.1; Table 4), Adding objects to the environment (BCTTv1 12.5; Table 4), Problem solving (BCTTv1 1.2; Table 4), Action planning (BCTTv1 1.4; Table 4), and Social support (practical) (BCTTv1 3.2; Table 4). These techniques are intended to reduce friction at the point of care by making reporting more visible, easier to initiate, and easier to complete within ordinary workflow. This is particularly important in high-pressure clinical environments, where even motivated staff may fail to report if the process is cumbersome or poorly integrated into daily work (Force et al., 2006; Abstoss et al., 2011).

Incentivisation was retained, but only cautiously and as a supporting rather than dominant intervention function. Recognition or reinforcement may help sustain reporting behaviour, especially where reporting has historically been deprioritised (Lehmann et al., 2007; Lederman et al., 2013; Massah et al., 2021). At the same time, incentivisation in this field carries an important risk: if applied inappropriately, it may shift attention from report quality and organisational learning to report volume alone. For this reason, MERP positions incentivisation as modest recognition linked to improvement and Feedback on outcome(s) of behaviour (BCTTv1 2.7; Table 4), rather than as a target-driven performance mechanism. This cautious positioning is also consistent with our decision to exclude coercive or intensive monitoring-oriented approaches that might increase fear, burden, or gaming.

The selected policy categories also contribute important implementation support and should be viewed as part of the intervention architecture rather than as peripheral additions. Communication/marketing can reinforce awareness and normalise non-punitive reporting messages; Regulation can clarify responsibilities, reporting procedures, and institutional protections; Service provision can support the practical infrastructure of reporting, including medication safety roles, reporting assistance, and technical support; and Fiscal measures may, where feasible, support modest recognition or implementation resources (Carandang et al., 2015). Together, these policy categories provide an implementation scaffold around the intervention functions and help explain how the behavioral components of MERP might be sustained in real-world hospital settings. As noted in the BCW framework, policy categories are not direct substitutes for BCTs, but broader levers that can support implementation and system alignment (Michie et al., 2011; Michie et al., 2014).

An important strength of this study is that BCTTv1 and TIDieR were used not as parallel or competing methods, but as sequential tools for intervention specification. In this study, the BCW identified the broad types of action needed to address the behavioral diagnosis, whereas BCTTv1 specified the intervention’s active ingredients, and TIDieR organised these into a deliverable protocol (Michie et al., 2013; Hoffmann et al., 2014). This sequencing is important because it improves transparency and reproducibility. Previous pharmacist-involved or pharmacist-led multicomponent interventions have often suggested promising effects, but the intervention-development process and active ingredients were not always clearly described (Force et al., 2006; Relihan et al., 2009). By contrast, MERP makes explicit how behavioral determinants were linked to intervention functions, how those functions were translated into specific BCTs, and how the resulting components were assembled into an implementable pharmacist-led programme.

The pharmacist-led nature of MERP is also noteworthy. Pharmacists are well positioned to support medication error reporting because their routine roles already intersect with medication review, reporting support, medication safety education, and system improvement. Prior studies suggest that pharmacist involvement can substantially strengthen medication safety and reporting performance. For example, pharmacist-led intervention and education have been associated with marked increases in medication error reporting and reductions in serious medication errors, and pharmacists often contribute substantially to the identification and reporting of medication errors (Costello et al., 2007; Samsiah et al., 2020; Gillani et al., 2021). In MERP, pharmacists are positioned not only as trainers, but also as coordinators of reporting support, workflow facilitation, feedback loops, and multidisciplinary learning. The present study therefore adds to the literature by specifying how a pharmacist-led intervention can be developed through an explicit behavioral and implementation logic.

Context is central to interpreting this intervention. Reporting behaviour in Chinese hospitals may be influenced by hierarchical structures, sensitivity around blame and reputation, workload burden, and medico-legal concerns, all of which may reduce the acceptability of directly importing interventions from other settings (Hu et al., 2024). MERP attempts to address this by combining diagnosis-driven intervention selection, contextual appraisal using APEASE, and stakeholder refinement of delivery arrangements. In this sense, the main contribution of the study is not to claim that one universal intervention has been identified, but to demonstrate a transparent method for developing a context-sensitive intervention blueprint.

A frequent limitation in behaviour change interventions is poor specification of “active ingredients” and delivery parameters, reducing replicability across sites. MERP addresses this by (i) defining component content using BCTTv1 and (ii) describing delivery using TIDieR (brief name, why, materials, procedures, who provides, how, where, when/how much, and planned fidelity), while noting that tailoring/modifications/actual fidelity will be documented during implementation (Hoffmann et al., 2014). This structured reporting should support subsequent piloting, process evaluation, and iterative optimisation.

Several limitations should be acknowledged. First, MERP remains at the intervention-development stage; its feasibility, acceptability, fidelity, effectiveness, and unintended consequences have not yet been empirically tested. Second, although the intervention was designed for Chinese hospitals, contextual variation across hospitals, departments, and professional groups may influence both implementation and response. Third, despite the structured process used here, component selection still involved interpretive judgement, particularly when determinants or candidate techniques overlapped conceptually. Fourth, no formal assessment of external validity or transferability was undertaken in this study.

For this reason, the wider applicability of the present work should be interpreted cautiously. Rather than claiming that MERP is already generalisable to other safety reporting behaviors, a more appropriate conclusion is that the intervention-development process used in this study may be transferable, in principle, to related reporting behaviors, provided that behavioral diagnosis, contextual appraisal, and intervention refinement are repeated in the new setting (Michie et al., 2014). Whether the resulting intervention content itself can be transferred across settings or behaviors remains an empirical question and requires formal evaluation.

Future work should therefore focus on piloting and process evaluation. In addition to reporting volume, evaluation should consider timeliness of reporting, near-miss capture, report completeness and quality, severity mix, feedback delivery, and evidence of organisational learning or corrective action. Balancing measures should include perceived blame, psychological safety, workload burden, and any unintended gaming effects, particularly where incentivisation is used. Such evaluation would help determine not only whether MERP works, but also which components are most influential, under what conditions, and with what implementation requirements.

## Conclusion

By integrating TDF/COM-B diagnosis with BCW-guided intervention mapping, this study produced a replicable, context-adapted MERP blueprint to strengthen medication error reporting in Chinese hospitals. MERP links behavioral determinants to feasible intervention functions and BCT-specified components, with delivery transparently described using TIDieR. This provides a robust foundation for pilot testing and subsequent hybrid effectiveness–implementation evaluation aimed at improving reporting-and-learning systems for medication safety.

## Data Availability

No new datasets were generated or analysed for this study. All materials supporting the intervention development (BCW mapping outputs, selected BCTs, and the TIDieR description of MERP) are provided in the manuscript and its supplementary material.
